# Lattice-Cluster-Theory-Informed Cross-Fractionation Chromatography Revealing Degree of Crystallinity of Single Macromolecular Species

**DOI:** 10.1021/acsmacrolett.4c00288

**Published:** 2024-07-30

**Authors:** Zengxuan Fan, Jana Zimmermann, Lucio Colombi Ciacchi, Michael Fischlschweiger

**Affiliations:** † Chair of Technical Thermodynamics and Energy Efficient Material Treatment, Institute for Energy Process Engineering and Fuel Technology, Clausthal University of Technology, Agricolastraße 4, 38678 Clausthal-Zellerfeld, Germany; ‡ Hybrid Materials Interface Group, Faculty of Production Engineering, Bremen Center for Computational Materials Science and MAPEX Center for Materials and Processes, University of Bremen, 28359 Bremen, Germany

## Abstract

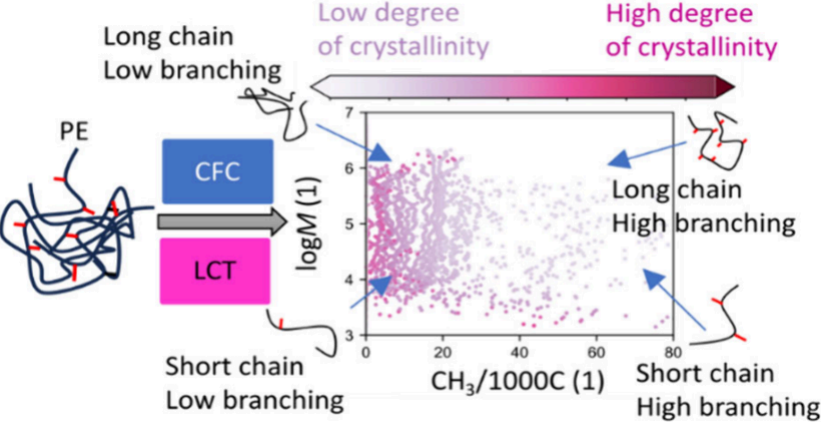

The relationship between macromolecular architecture and crystallization properties is a relevant research topic in polymer science and technology. The average degree of crystallinity of disperse polymers is a well-studied quantity and is accessible by various experimental methods. However, how the different macromolecular species contribute to the degree of crystallinity and, in particular, the relationship between a certain macromolecular architecture and the degree of crystallinity are not accessible today, neither experimentally nor theoretically. Therefore, in this work, a lattice cluster theory (LCT)-informed cross-fractionation chromatography (CFC) approach is developed to access the degree of crystallinity of single and nonlinear macromolecular species crystallizing from solution. The method entangles high-throughput experimental data from CFC with the LCT for semicrystalline polymers to predict the degree of crystallinity of polymer species with different molecular weights and branching. The approach is applied to a linear low-density polyethylene (ethylene/1-octene copolymer) and a high-density polyethylene, which have specific and different bivariate distributions. The degree of crystallinity of individual macromolecular species of these polymer samples is calculated, and the predicted average degree of crystallinity is compared with experimental measurements, thus successfully validating the approach. Furthermore, the average segment length between branches is introduced as a characteristic molecular feature of branched polyethylene, and its relationship with the degree of crystallinity of certain species is established.

Crystallization of polymers out of the bulk and out of solution is a highly relevant research topic from today’s perspective because various key phenomena of polymer crystallization are not fully understood yet.^1^ This particularly includes the relationship of macromolecular architecture and crystallization in equilibrium as well as under the influence of internal and external kinetic effects, which additionally strongly triggers polymer morphology formation.^2−4^ In previous studies, a wide range of methods have been developed to determine mesoscopic morphology characteristics such as the degree of crystallinity by applying wide-angle/small-angle X-ray diffraction (WAXS/SAXS),^5,6^ density-based measurements,^7^ differential scanning calorimetry (DSC),^8−10^ infrared spectroscopy (IR),^11^ Raman spectroscopy (Raman),^12^ and nuclear magnetic resonance spectroscopy (NMR).^13,14^ Such measurements are applied in bulk and in solution and give access to an intermediate degree of the crystallinity of the polymer. Furthermore, Raman spectroscopy has successfully revealed nonhomogeneous spatial distribution of the degree of crystallinity within the bulk of a polymer. Experimental observations show that crystalline structures are also spatially nonuniformly distributed, which can be attributed to spatial differences of dispersity in molecular weight and branching, kinetic effects, and local micromechanical interactions.^15−17^ Additionally, molecular dynamics simulations of simple polymer model systems have revealed that crystallization is strongly influenced by molecular features such as chain entanglement^18^ and branching.^19^ In particular, the crystallization kinetics was observed to be influenced by branching content but not by branch length, which however affected the obtained crystal morphologies.^20^

This underpins the complexity of understanding polymer crystallization. One crucial partial aspect is the relation between molecular architecture in terms of molecular weight and branching, e.g., branching in polyethylene, with a particular degree of crystallinity formed out of the bulk or out of solution under quasi-equilibrium conditions. Hence, the interrelation between the morphological characteristic, “degree of crystallinity”, with a specific polymer architecture, i.e., single value of molecular weight and branching referring to a certain macromolecular species, is currently inaccessible. However, gaining access to this relationship could be a key factor in enhancing molecular design capabilities within the context of engineering semicrystalline morphologies. If this relation can be established, a property-originated design can be carried out by controlling architecture with sophisticated fractionation^21^ or synthesis methods.^22−24^ A complex distribution of molecular architectural features (molecular weight and branching) occurs, for instance, in polyethylene. Understanding this complexity is key in terms of establishing tailor-made properties. However, at the same time, it is a challenging research topic. Additionally, obtaining information that establishes connections between molecular architectural features and the degree of crystallinity of particular macromolecular systems requires new and efficient information-acquiring strategies that can gain this information in a timely and convenient manner. Furthermore, this is important for advancing thermodynamic as well as data-driven crystallization modeling strategies, which would also contribute to the Polymer Genome^25^ initiative.

This work aims to develop a method that provides access to the relationship between single macromolecular species, showing a monodisperse molecular architecture, i.e., molecular weight and branching, and the degree of crystallinity, where here the focus is put on solution crystallization. To achieve this goal, in this work, a novel approach to directly couple high-throughput multidimensional chromatography with a statistical thermodynamic model framework is developed.

The multidimensional chromatography applied in this work is cross fractionation chromatography (CFC). This method is based on the in situ combination of temperature-rising elution fractionation (TREF) and size exclusive chromatography (SEC)^26,27^ in the TREFxSEC mode. The detailed working principle is sketched in the Supporting Information and further described in ref (22). The analysis is carried out under very low cooling and heating rates (0.1–0.5 K/min), such that each TREF step can be assumed to occur in a quasi-equilibrium state. Therefore, the statistical modeling approach can be carried out under the equilibrium conditions. CFC typically provides a bivariate distribution of molecular weight (MWD) and branching (*b*) of a polymer as well as the solid–liquid temperature *T*^LS^ for certain macromolecular species crystallizing out of solution at a certain concentration in a comparably short time period, which is around 24–48 h. The experimentally obtained large data set is initially composed of the vector (*MW*, *b*, *T*^SL^)_*i,j*_ for each macromolecular species *i,j* (monodisperse fraction). The data set serves consequently as input for the statistical thermodynamic model, which allows us to access the degree of crystallinity and will be explained in the forthcoming paragraph. The index *i* corresponds to the experimentally introduced TREF intervals, and *j* points to respective SEC intervals.

Understanding and predicting the degree of crystallinity directly from molecular architecture out of solution is an active and challenging research field.^28^ First statistical thermodynamic models which are based on the lattice cluster theory (LCT)^29−33^ have been developed in the past to predict solid–liquid equilibria (SLE) of polymer solvent systems, where molecular architecture is directly taken into account and semicrystallinity combined with dispersity of the polymer is thermodynamically consistently considered.^21,34−41^ This work relies on the respective model framework and applies a pseudo component-based approach to treat dispersity. Co-crystallization effects between two different macromolecular species are not considered, due to the dilute solution crystallization in CFC.^42^ Hence, no interactions between the macromolecular species treated in the model framework as pseudo components need to be considered. TREF delivers *n* fractions, and SEC delivers *m* sampling points. The thermodynamic equilibrium condition for the SLE can be written on the macromolecular species level:
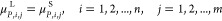
1where μ_*P*,*i*,*j*_^L^ is the chemical potential of pseudo components *i,j* of the polymer in the liquid phase, and μ_*P*,*i*,*j*_^S^ is the chemical potential of pseudo components *i,j* of the polymer in the solid phase. Each pseudo component corresponds to a certain macromolecular species, showing a particular molecular weight (*MW*) and branching (*b*).

The segment-molar fraction φ_*P*,*i*,*j*_ of the macromolecular species *i,j* in the solution can be calculated by
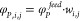
2where φ_*P*_^feed^ is the segment-molar fraction of the whole polymer in the solvent, and *w*_*i*,*j*_ is the segment-molar fraction of the macromolecular species *i,j*, directly obtained from CFC measurements. Furthermore, branching is measured for each macromolecular species via NMR-calibrated IR5 detection in CFC. The IR detector calibration for detecting the branching number is executed based on the measurement of six ethylene/1-octene standard materials. The standards were characterized in terms of branching number with ^13^C NMR and were purchased from Polymer Char. More detailed information about the calibration standards is given in the Supporting Information. Based on this, for each macromolecular species now the following information is available as model input (*r*, *b*, *φ*_*P*_, *T*^LS^)_*i,j*_, where *r* is the segment number; *b* is the branching number; *φ*_*P*_ is the segment-molar fraction; and *T*^LS^ is the solid–liquid transition temperature. Applying the already developed LCT framework^37^ to predict the SLE of polymer solvent systems, where the polymer behaves as semicrystalline, with a degree of crystallinity of (1 – λ), where λ is the amorphization ratio, in combination with eqs 1 and 2, leads to the final working equation which couples LCT with CFC input directly and gives access to the degree of crystallinity:
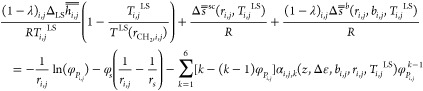
3In eq 3,  is the segment-molar enthalpy of fusion of the bulk polymer; *T*^LS^(*r*_CH_2_,*i*,*j*_) is the solid–liquid temperature of the bulk polymer, known for polyethylenes from correlation functions; and *r*_CH_2_,*i*,*j*_ is the segment length of species *i*,*j* in terms of the number of methylene groups.^22^and  are entropic correction terms due to the specific molecular architecture; *φ*_*s*_ is the segment-molar fraction of the solvent *s*; and α_*i*,*j*,*k*_ is a molecular architecture coefficient associated with a particular macromolecular species *i,j* and depends on the lattice coordination number *z*, on the interaction energy Δε between solvent and polymer species, as well as on *b* and *r* of the molecular species. Expressions for molecular architecture coefficients α_*i*,*j*,*k*_ can be derived based on the LCT framework.^29−33^ Derivations and architecture coefficients applied in this work are given in refs (38 and 43−48). Detailed expressions for and  are given in refs (37, 39, and 40). The determination of the interaction parameter of the polymer and solvent Δε is carried out according to ref (9).

Because the quantities (*r*, *b*, *φ*_*P*_, and *T*^LS^)_*i,j*_ are experimental input from CFC, the only remaining unknown is the macromolecular species specific degree of crystallinity (1 – λ)_*i*, *j*_, which can then be determined by solving eq 3 for each species. This demonstrates the direct coupling of LCT with CFC to access the macromolecular species-specific degree of crystallinity out of solution.

A mean value of the degree of crystallinity of the polymer (1 – λ) can be calculated with the weighted sum of the degree of crystallinity of each macromolecular species and their segment-molar fraction:
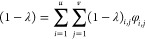
4This newly introduced method of LCT-informed CFC to access the degree of crystallinity of single macromolecular species out of solution crystallization is now applied to two different polyethylene samples, LLDPE and HDPE, respectively. The detailed material’s descriptions are given in the Supporting Information. 1,2,4-Tricholorobenzene (TCB) is used as the solvent. The CFC method started with first heating the polymers in TCB to 160 °C at a concentration of 2 mg mL^–1^ and then cooling to room temperature before it undergoes multistep heating. Between the two temperatures, the partly dissolved polymer is eluted with solvent and is injected into the SEC columns, to measure the molecular weight distribution (MWD) of the respective fraction. In the cross-fractionation process, *n* was set to 24 and *m* to 241. A more detailed description of the TREFxSEC applied to these samples is given in the Supporting Information.

The obtained distributions given here in log(*M*) over *T*^LS^ are presented for LLDPE (ethylene/1-octene copolymer) in Figure 1(a) and HDPE in Figure 1(b).

**Figure 1:**
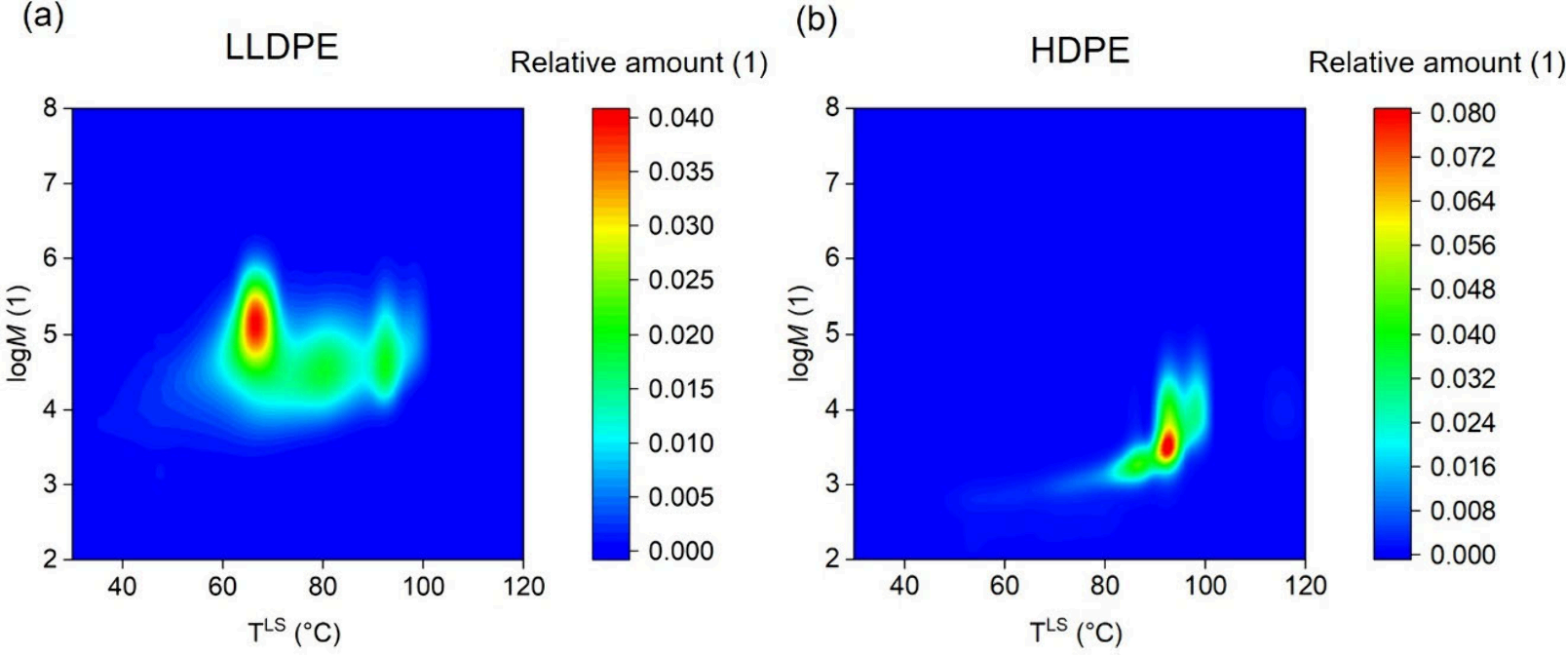
CFC bivariate distribution showing a relative amount of the macromolecular species in (a) for LLDPE and (b) for HDPE. The intensity of color reflects the relative amount according to the given legend.

These different molecular architectures show a broad variation and high number of different macromolecular species, which are hence well suited for testing the LCT-informed CFC approach to evaluate the degree of crystallinity distribution for a broad molecular architecture distribution. By applying eq 3 and inserting the information obtained from CFC for those two example polymers, (1 – λ)_*i*,*j*_ for LLDPE and for HDPE are gained and visualized in Figure 2(a) and 2(b).

**Figure 2:**
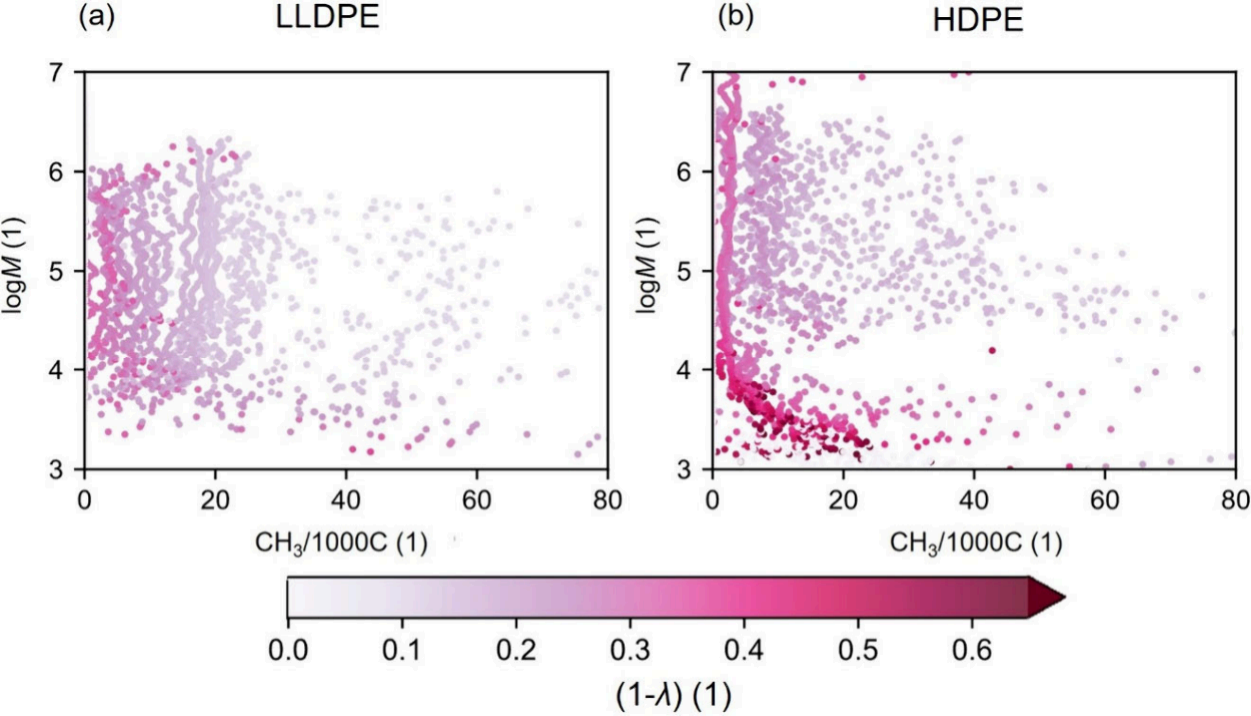
Degree of crystallinity of macromolecular species for two different polyethylene types: (a) LLDPE and (b) HDPE with respect to the molecular weight (log *M*) and branching given by CH_3_/1000C, where a deeper color indicates a higher degree of crystallinity.

Figure 2 shows a pointwise two-dimensional projection of (1 – λ)_*i*,*j*_ in the molecular weight and branching plane, where branching is given in Figure 2 as the number of CH_3_ groups per 1000C atoms in the backbone. The deeper the color, the higher the value of (1 – λ)_*i*, *j*_ exhibited by a certain macromolecular species *i,j*. For both polymers investigated with the newly developed method, a low branching number of a specific species in combination with a log(*M*) > 4.5 leads to high degrees of crystallinities. The degree of crystallinity gradually decreases when branching number increases. In the molecular weight region log(*M*) < 4.5, the behavior is different. On one hand, when the molecular weight of the respective species is low, the degree of crystallinity is comparably high even with increasing branching numbers. On the other hand, the changes in the degree of crystallinity with increasing branching number are significantly more pronounced.

Therefore, a nonlinear correlation between molecular weight, branching, and degree of crystallinity is found. Assuming that the degree of crystallinity of the macromolecular species has a certain relationship with the crystalline sequence length, which is defined herein as “average segment length between branches” and denoted by η, the feature given in eq 5 is introduced for developing respective correlations between this molecular feature and the degree of crystallinity:
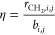
5With the measure defined in eq 5, the relationship with the degree of crystallinity gained by the LCT-informed CFC approach is investigated for molecular weights above and below log(*M*) = 4.5. Corresponding results are shown in Figure 3(a) and (b) for LLDPE and HDPE, respectively. Our general expectation would be that the degree of crystallinity increases with increasing average segment length between branches. In fact, for macromolecular species above log(*M*) = 4.5 the relationship between degree of crystallinity and log(η) is almost linear for both LLDPE and HDPE (Figure 3c) and independent of the molecular weight. However, strong differences appear in the gained data for molecular weights below log(*M*) = 4.5. In this case, nonlinearities appear, and the degree of crystallinity is much higher compared to longer molecules with the same values of η.

**Figure 3:**
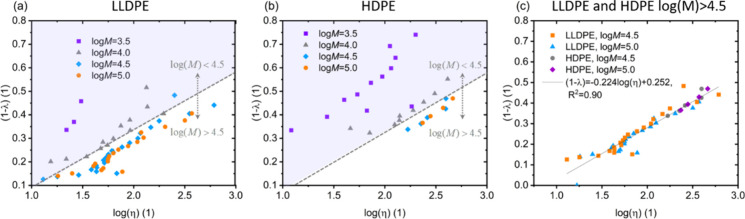
(a) Degree of crystallinity over the logarithm of average segment length between branches for LLDPE and certain molecular weight fractions. (b) Degree of crystallinity of the logarithm of average segment length between branches for HDPE and certain molecular weight fractions. (c) Linear regression of the species of LLDPE and HDPE with log(*M*) = 4.5 and 5.0.

We could speculate that the degree of crystallinity might be highly dependent on the branch length. Below a critical molecular weight, the shortness of the branches may allow for better accommodation of branching defects between well-developed crystalline regions. On the contrary, longer branches may frustrate crystal growth either by competing nucleation events (a kinetic effect) or by more favorable entropy of large amorphous regions (a thermodynamic effect). These nonlinear relations below a certain molecular weight seem to be an interesting topic to be investigated in the future. Moreover, the correlations for log(*M*) < 4.5 are less clear than for log(*M*) > 4.5, which could be related to possible deviations from the average segment length in nonuniform branching sequences. This particularly motivates further research of crystallization mechanisms with short chains and broad branching distributions.

For the sake of comparison and to validate the present approach on a macroscopic scale, the calculated mean value of the degree of crystallinity of both polymers obtained by eq 4 is compared with the measured degree of crystallinities of the polymer solutions via DSC (differential scanning calorimetry). Details regarding the applied method are listed in the Supporting Information. The results show that the predicted mean value of the degree of crystallinity (eq 3) is 0.27 for LLDPE and 0.44 for HDPE. The experimental values are 0.28 for LLDPE and 0.45 for HDPE, respectively. This shows a very good comparison between experimental and calculated mean values of the degree of crystallinity for these particular samples, overall. In order to validate the approach also at the fractional level, Column-based Preparative (PREP) Fractionation (PREP C20) in TREF mode was used to obtain fractions with a comparably narrow molecular weight and branching distributions with respect to the parent sample. The two fractions (fractions 1 and 2) have a number-average molecular weight *M*_*n*_ of 18,795 g mol^–1^ (fraction 1) and 25,393 g mol^–1^ (fraction 2). The branching number *b* of fraction 1 is 20.8 CH_3_ 1000C^–1^, and that of fraction 2 is 8.5 CH_3_ 1000C^–1^. Detailed distributions of the respective fractions are given in the Supporting Information. The same procedure is carried out to measure the degree of crystallinity as above, where a value of 0.19 and 0.35 is determined for fractions 1 and 2, respectively. Applying eq 3 gives the predicted values for the degree of crystallinity of 0.22 and 0.35, respectively. This shows good agreement between the prediction and the experimentally obtained values, further validating the approach at the fraction level.

In addition to the degree of crystallinity, this approach gives access to MWD transitions in the respective solid and liquid phases due to the temperature in certain lower-molecular weight regimes. For demonstration purposes, the MWD of HDPE in the liquid phase at a single temperature, 361 K, is predicted with the LCT-based approach, using now the degree of crystallinity distribution obtained with the LCT-informed CFC approach. The result is shown in Figure 4, and the measured MWD in the liquid phase of HDPE at 361 K in TCB with the same concentration used in CFC is compared with the predicted MWD via LCT. To compare the measured and predicted MWD with the feed MWD, the gray curve is presented in Figure 4. With this calculation procedure, the MWD of the polymer in the liquid phase having a certain degree of crystallinity could be predicted in very good agreement with experimental observations. This shows the potential of coupling LCT-based modeling approaches with high-throughput chromatography also in terms of tailored polymer separations.

**Figure 4:**
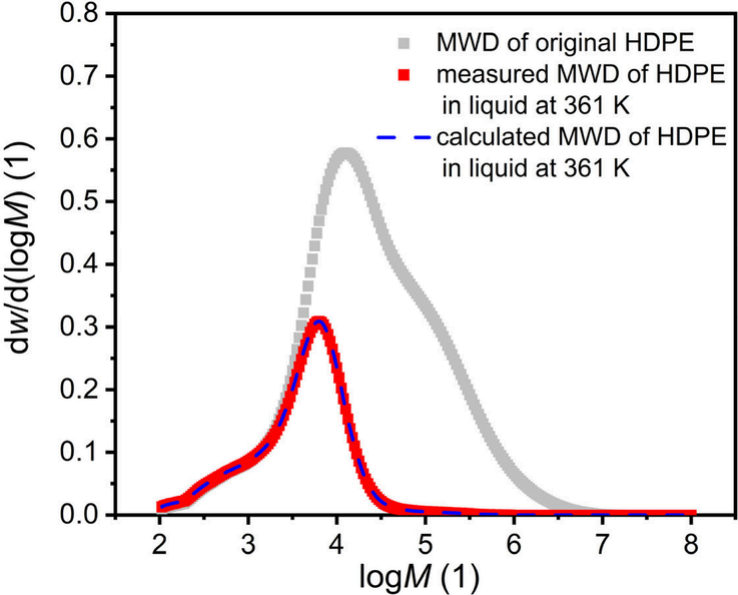
Measured and calculated MWD of HDPE in the liquid phase at 361 K in combination with the MWD of feed HDPE. The measured MWD of the original HDPE is presented in gray squares; the MWD of polymer fraction remaining in the liquid phase when fractionated at 361 K is stated with red squares; and the calculated MWD of polymer remaining in the liquid phase when fractionated at 361 K with varying degree of crystallinity for different polymer species is given via blue dash lines.

In conclusion, this work presents a novel method of LCT-informed CFC to access the degree of crystallinity of single macromolecular species in solution crystallization under equilibrium conditions. The method combines the high-throughput experimental data from CFC with the statistical thermodynamic model based on LCT to predict the degree of crystallinity of different polymer species with respect to their molecular weight and branching. The method was applied to two polyethylene samples, LLDPE and HDPE, and showed good agreement with the DSC measurements and the MWD of the liquid phase fractions. The method also revealed some interesting insights into the influence of molecular architecture on the degree of crystallinity and particularly molecular weight driven nonlinearity effects. Hence the feature of average segment length between branches is introduced, and the relation to the degree of crystallinity of single macromolecular species could be established for the first time, where a linear relationship between degree of crystallinity and log(η) is found above a molecular weight of log(*M*) = 4.5, independent for the polymers investigated. This method contributes to the further understanding of polymer crystallization based on certain macromolecular architecture features.

## Supporting Information Available

Information on materials, description of experimental methods and calibration procedure, and validation of the approach at the fractional level (PDF)
