# Supplementary material for: Lattice-Cluster-Theory-Informed Cross-Fractionation Chromatography Revealing Degree of Crystallinity of Single Macromolecular Species
Source: ACS Macro Lett. 2024 Jul 30;13(8):1043–9. doi: 10.1021/acsmacrolett.4c00288 (PMC11340020; doi:10.1021/acsmacrolett.4c00288)
Supplement: Supplementary file 1 — mz4c00288_si_001.pdf [file mz4c00288_si_001.pdf]

Supporting Information

Lattice-Cluster-Theory-Informed Cross-Fractionation Chromatography Revealing Degree of Crystallinity of Single Macromolecular Species

*Zengxuan Fan[1), Jana Zimmermann[1), Lucio Colombi Ciacchi[2), Michael Fischlschweiger[1)**

[1)Chair of Technical Thermodynamics and Energy Efficient Material Treatment, Institute for Energy Process Engineering and Fuel Technology, Clausthal University of Technology, Agricolastraße 4, 38678 Clausthal-Zellerfeld, Germany

[2)Hybrid Materials Interface Group, Faculty of Production Engineering, Bremen Center for Computational Materials Science and MAPEX Center for Materials and Processes, University of Bremen, 28359 Bremen, Germany

*Corresponding author email address: michael.fischlschweiger@tu-clausthal.de

Materials

In this work, 1,2,4-Trichlorobenzene (TCB) (99 %) from Sigma-Aldrich (SAL.132047) is used as solvent to dissolve respective PE samples. Methanol is used as an antisolvent. It was purchased from Sigma-Aldrich (SAL.322415) with a purity of 99.8 %. The ethylene/1-octene copolymer referred to as LLDPE was provided by Polymer Char, where detailed molecular characterization information is provided in Table 1. The high-density polyethylene referred to as HDPE from Sigma-Aldrich (SAL.547999) is also characterized and results are provided in Table S1.

Table S1 Molecular Characterization of LLDPE and HDPE.

Polymer	LLDPE	HDPE
M_w (g mol^{-1})	85057	112148
M_n (g mol^{-1})	23074	4583
CH_3 1000C^{-1}*	14.1	5.0
*(1-λ)***	0.28	0.47

* average number of CH_3 per 1000 Carbon atoms referred to as *b* in the manuscript. ** average degree of crystallinity of respective polymer measured by DSC in this work.

Experimental Methods

Cross Fractionation Chromatograph (CFC)

To investigate bivariate distribution of polyethylene, Cross Fractionation Chromatograph (CFC) is utilized to separate the polymer into different fractions. CFC is an automated instrument and has been developed for measuring the bivariate distribution by TREF fractionation and subsequent SEC analysis of the fractions in a single run.[1] A sketch of the basic CFC components is provided in Figure S1.

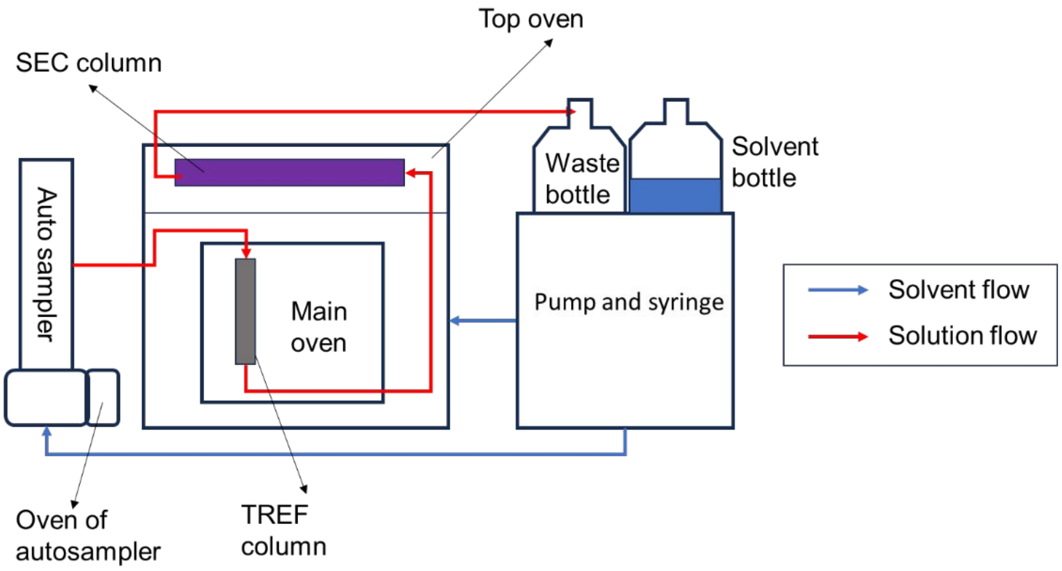

Figure S1 A schematic diagram of the major components of CFC.

The measurement process of all samples given in CFC in this work is given as follows: First 16 mg of polymer sample is weighted and taken into the auto sampler vial (Figure S1), where 8 ml 1,2,4-TCB is injected with an automated syringe. This mixture is then heated up to 160 °C and held for 60 min, so that a 2 mg ml^{-1} solution is formed and due to the low concentration co-crystallization

is inhibited. Subsequently, 1 ml of this solution is injected into the TREF column for further analysis. In the TREF column, the sample is separated mainly according to branching with the temperature method given in Figure S2.

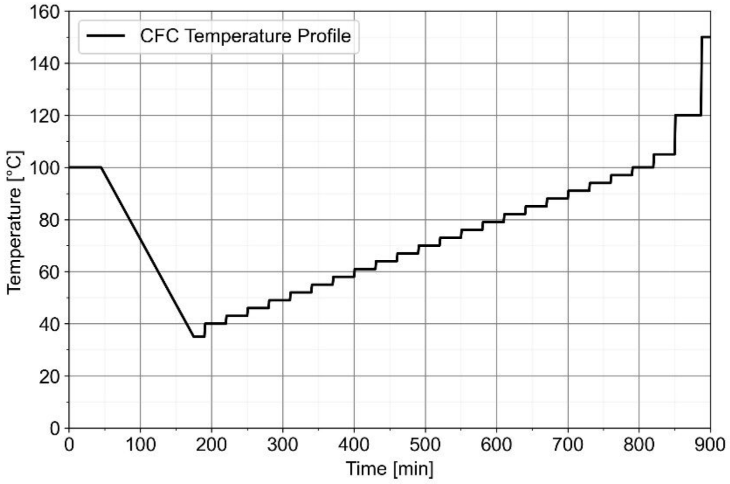

Figure S2 CFC temperature profile applied in the multistep fractionation process.

In the first TREF step, the column temperature is kept for stabilization at 100 °C for 45 min. To crystallize the polymer sample, the temperature is cooled down with a rate of 0.5 °C min^{-1} to 35 °C and stabilized at that temperature for 15 min. Due to the low cooling rate, it can be assumed that quasi-equilibrium conditions are reached, and co-crystallizations and kinetic effects are inhibited. In order to obtain the different fractions, the temperature is raised stepwise with temperature steps of 3 °C at a heating rate of 20 °C min^{-1} between the temperature interval of 35 °C and 100 °C. The last three fractions are eluted at 105 °C,120 °C and 150 °C, so that in total the polymer is fractionated into 24 fractions. At each temperature step, the sample is held for 25 min to ensure the dissolution of the polymer fraction with a certain chemical composition. The separation from the remaining sample is then achieved by an elution in a time interval of 5 min. To determine the

molecular weight distribution of the TREF-fraction, the eluted fractions are pumped into the SEC columns where it is eluted at 1 ml min^{-1}.

The amount of different polymer species in each fraction eluted is determined with an IR5 detector. IR signals against retention time for the polymer that is fractionated at different temperatures are provided for LLDPE and HDPE Figure S3a and b, respectively.

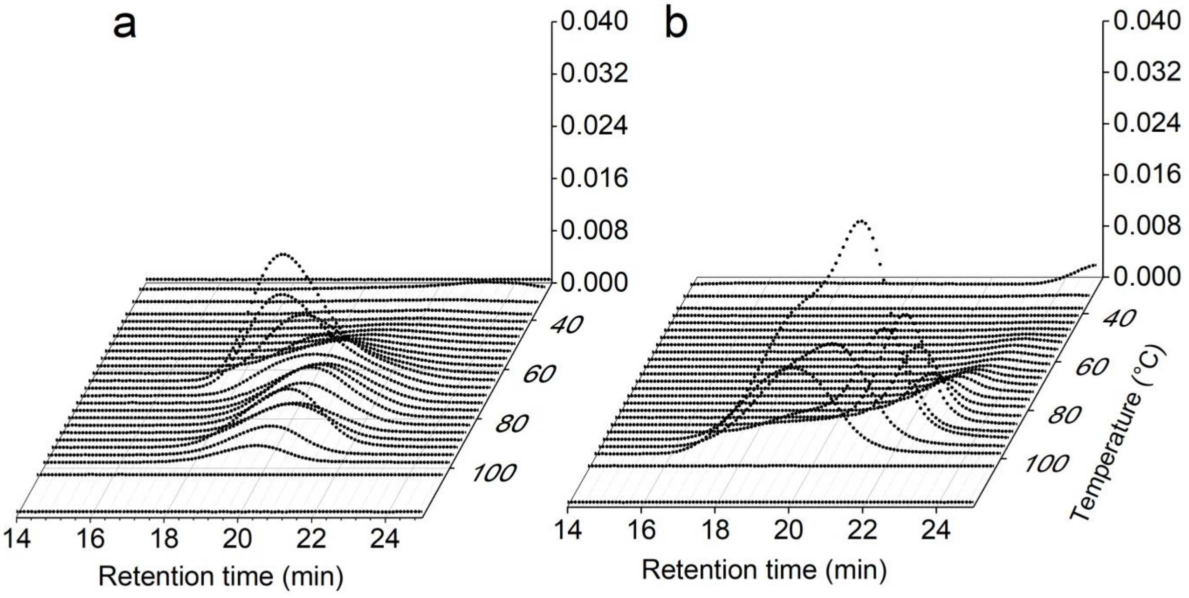

Figure S3 IR5 detector signals of cross-fractionation (a) of LLDPE and (b) of HDPE

The calibration of the SEC column was carried out by PS and PE samples provided by Polymer Char as standard, where the following Mark-Houwink parameters for PE, K=0.0004416, α=0.725 and for PS, K=0.0001020, α=0.722 are applied.

Furthermore, the branching is obtained by the specific IR signals of the CH_3 groups. The relation of the IR signal with the CH_3 groups is obtained through a calibration with six ethylene/1-octene copolymer standards from Polymer Char. These standards have been characterized in terms of

branching number with ^{13}C-NMR by Polymer Char. The characterization information of the standards is provided in Table S2.

Table S2 Information of the ethylene/1-octene copolymer standards provided by Polymer Char

Standard name	CH_3 1000C^{-1}	Comonomer content (wt%)	Density (g cm^{-3})
O-1	2.6	2.1	0.940
O-2	16.5	13.2	0.909
O-3	21.0	16.8	0.902
O-4	33.5	26.8	0.885
O-5	45.3	36.2	0.868
O-6	45.9	36.7	0.870

Column-based Preparative (PREP) Fractionation (PREP C20)

To validate the new approach at the fractional level, two fractions from the LLDPE sample have been extracted from the parent sample with Column-based Preparative Fractionation (PREP C20) working in TREF-mode. The apparatus was purchased from Polymer Char. The experimental temperature profile of the experiment is identical with the procedure regarding the CFC experiment. Therefore, 3 g of LLDPE are placed into a vessel and dissolved in 1,2,4-TCB at 160 °C for 2 hours. The solution is then pumped into a TREF column, where the solution is cooled to 35°C and held for 10 minutes for stabilization. The temperature is raised to a certain elution temperature, and the solvent is pumped through the column. Two fractions, collected at 67 °C and 91 °C, are physically obtained and are referred to as fraction 1 and fraction 2 in the following paragraphs.

The obtained fractions are concentrated at 160 °C and 140 mbar using a rotary evaporator for solvent recovery. The concentrated polymer solution is then precipitated with methanol as an antisolvent. After stabilizing for 2 hours, the solid is filtered through a 0.45 µm polytetrafluoroethylene membrane. The polymer is recovered from the filter and dried in a vacuum oven at 50 °C for 12 hours to remove any residual solvent and antisolvent. The bivariate characterization by CFC of fraction 1 and fraction 2 is presented in Figure S4 and S5.

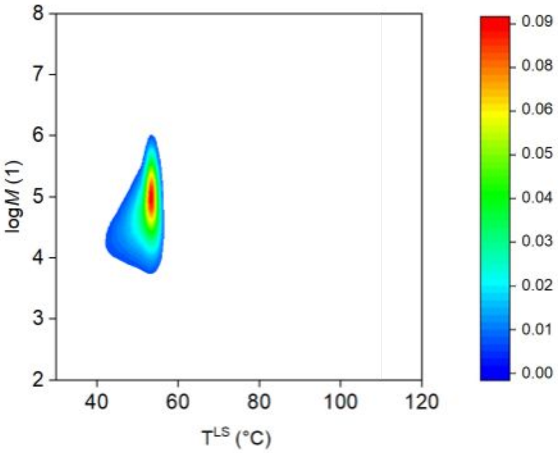

Figure S4 CFC bivariate distribution showing relative amount of the macromolecular species in LLDPE fraction 1 obtained at 67 °C. The intensity of color reflects the relative amount quantity according to the given legend.

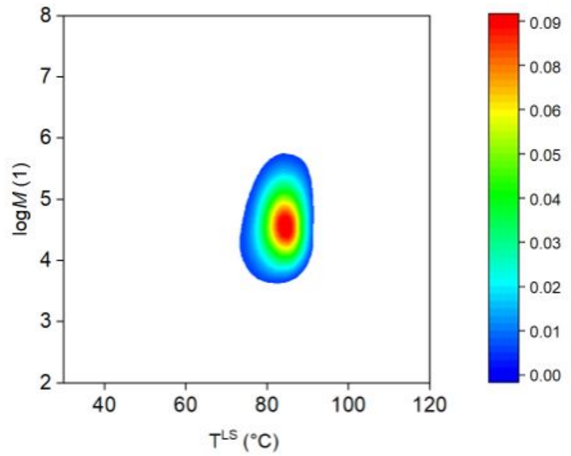

Figure S5 CFC bivariate distribution showing relative amount of the macromolecular species in LLDPE fraction 2 obtained at 91 °C. The intensity of color reflects the relative amount quantity according to the given legend.

Fraction 1 and fraction 2 are then measured with DSC according to the description in the respective part.

Differential scanning calorimetry (DSC)

DSC measurements are carried out to measure the melting enthalpy of the PE samples and the fractions. The results will be used to determine the average degree of crystallinity and will be compared to the results calculated for a validation purpose.

Therefore, a Calvet HT DSC (differential scanning calorimeter) from Setaram KEP Technologies SA is applied. In this DSC, to detect the heat flow in three dimensions, a thermopile sensor consisting of 144 thermocouples was utilized. This sensor was calibrated using the Joule effect calibration method. Additionally, the cells were temperature calibrated using standard substances such as indium, zinc, bismuth, and tin, following IUPAC guidelines. For each standard substance,

three heating cycles were performed, with the first measurement being disregarded to ensure reproducibility. In order to obtain a calibration that is independent of the heating rate, measurements were conducted at different rates (0.1, 0.3 and 0.5 K min^{-1}) and compared with measurements taken at 1 K min^{-1}.

Before conducting the DSC experiment, the samples undergo the same heating and cooling process in 1,2,4-TCB solution at identical concentration as in the CFC analysis program. This ensures a comparability between the results of the DSC experiments and the new LCT-informed CFC approach. The samples are then filtered and dried at room temperature at a pressure of 200 mbar for 3 days. A sample from 68.4 mg of LLDPE and 74.5 mg HDPE is measured with the Calvet HT DSC at a heating rate of 1 K min^{-1}. DSC signals are provided in Figure S6.

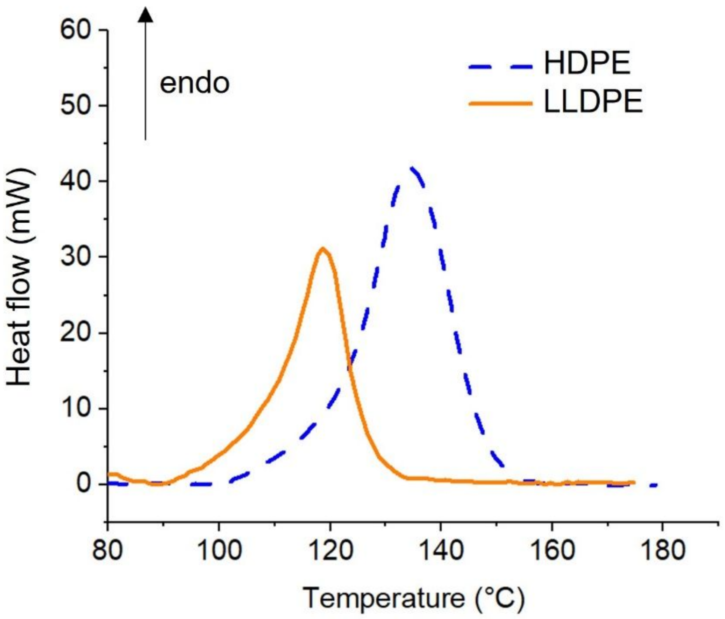

Figure S6 DSC curves of HDPE and LLDPE

For a validation on the fractional level, the degree of crystallinity of fraction 1 and fraction 2 from LLDPE is going to be measured with DSC. The sample goes through the same pre-treatment as the parent sample. Then they are measured with DSC under 1 K min^{-1}.

References

1. Ortin, A.; Monrabal, B.; Sancho-Tello, J. In Development of an Automated Cross-Fractionation Apparatus (TREF-GPC) for a Full Characterization of the Bivariate Distribution of Polyolefins, *Macromolecular symposia* **2007**, 257, 1, 13-28.
